# Accuracy of point-of-care testing for circulatory cathodic antigen in the detection of schistosome infection: systematic review and meta-analysis

**DOI:** 10.2471/BLT.15.158741

**Published:** 2016-04-22

**Authors:** Anthony Danso-Appiah, Jonathan Minton, Daniel Boamah, Joseph Otchere, Richard H Asmah, Mark Rodgers, Kwabena M Bosompem, Paolo Eusebi, Sake J De Vlas

**Affiliations:** a Department of Epidemiology and Disease Control, School of Public Health, University of Ghana, PO Box LG13, Legon, Ghana.; b School of Social and Political Sciences, University of Glasgow, Glasgow, Scotland.; c Department of Microbiology, Centre for Plant Medicine Research, Mampong, Ghana.; d Department of Parasitology, University of Ghana, Legon, Ghana.; e School of Biomedical and Allied Health Sciences, University of Ghana, Accra, Ghana.; f Centre for Reviews and Dissemination, University of York, York, England.; g Health Planning Service, Regional Health Authority of Umbria, Perugia, Italy.; h Department of Public Health, Erasmus University Medical Centre Rotterdam, Rotterdam, Netherlands.

## Abstract

**Objective:**

To assess the accuracy of point-of-care testing for circulatory cathodic antigen in the diagnosis of schistosome infection.

**Methods:**

We searched MEDLINE, EMBASE, LILACS and other bibliographic databases for studies published until 30 September 2015 that described circulatory cathodic antigen testing compared against one to three Kato–Katz tests per subject – for *Schistosoma mansoni* – or the filtration of one 10-ml urine sample per subject – for *S. haematobium*. We extracted the numbers of true positives, false positives, true negatives and false negatives for the antigen testing and performed meta-analyses using a bivariate hierarchical regression model.

**Findings:**

Twenty-six studies published between 1994 and 2014 met the inclusion criteria. In the detection of *S. mansoni*, a single antigen test gave a pooled sensitivity of 0.90 (95% confidence interval, CI: 0.84–0.94) and a pooled specificity of 0.56 (95% CI: 0.39–0.71; *n* = 7) when compared against a single Kato–Katz test. The corresponding values from comparisons with two to three Kato–Katz tests per subject were 0.85 (95% CI: 0.80–0.88) and 0.66 (95% CI: 0.53–0.76; *n* = 14), respectively. There appeared to be no advantage in using three antigen tests per subject instead of one. When compared against the results of urine filtration, antigen testing for *S. haematobium* showed poor sensitivity and poor specificity. The performance of antigen testing was better in areas of high endemicity than in settings with low endemicity.

**Conclusion:**

Antigen testing may represent an effective tool for monitoring programmes for the control of *S. mansoni*.

## Résumé

**Objectif:**

Évaluer la précision des tests de détection de l'antigène cathodique circulant pratiqués sur les lieux des soins pour le diagnostic des schistosomiases.

**Méthodes:**

Nous avons fait des recherches dans MEDLINE, EMBASE, LILACS et d'autres bases de données bibliographiques pour trouver des études publiées jusqu'au 30 septembre 2015 décrivant les résultats de tests de détection de l'antigène cathodique circulant comparativement à ceux obtenus avec un, deux ou trois tests Kato-Katz réalisé(s) pour chaque sujet (pour le dépistage d'une infection à *Schistosoma mansoni*) ou comparativement aux résultats de l’examen par filtration d'un échantillon de 10 ml d'urine par sujet (pour le dépistage d'une infection à *S. haematobium*). Nous avons extrait les nombres de vrais positifs, de faux positifs, de vrais négatifs et de faux négatifs associés à la technique de détection antigénique et nous avons réalisé des méta-analyses en employant un modèle de régression hiérarchique bivarié.

**Résultats:**

Vingt-six études publiées entre 1994 et 2014 ont respecté les critères d'inclusion. Pour le dépistage des infections à *S. mansoni*, avec un test unique de détection antigénique, nous avons obtenu une sensibilité combinée de 0,90 (intervalle de confiance -IC- de 95%: 0,84–0,94) et une spécificité combinée de 0,56 (IC de 95%: 0,39–0,71; *n* = 7) par comparaison avec les résultats d'un seul test Kato-Katz. Par comparaison avec les résultats de deux ou de trois tests Kato-Katz pour un même sujet, ces valeurs ont été estimées à 0,85 (IC de 95%: 0,80-0,88) et 0,66 (IC de 95 % : 0,53–0,76; *n* = 14) respectivement. La réalisation de trois tests de détection antigénique plutôt que d'un seul ne semble apporter aucun avantage. Pour le dépistage des infections à *S. haematobium*, les tests de détection antigénique se sont révélés peu sensibles et peu spécifiques par comparaison avec les résultats des examens d'urine par filtration. Les tests de détection antigénique ont été plus performants dans les régions à forte endémicité que dans les régions à faible endémicité.

**Conclusion:**

Les tests de détection antigénique peuvent constituer des outils efficaces pour le suivi des programmes de lutte contre *S. mansoni*.

## Resumen

**Objetivo:**

Evaluar la exactitud de las pruebas en el punto de atención en busca de antígenos catódicos circulantes en el diagnóstico de infección por esquistosomas.

**Métodos:**

Se realizaron búsquedas en MEDLINE, EMBASE, LILACS y otras bases de datos bibliográficas para encontrar estudios publicados hasta el 30 de septiembre de 2015 que describiesen las pruebas de antígenos catódicos circulantes en comparación con entre una y tres pruebas de Kato–Katz por sujeto (para encontrar *Schistosoma mansoni*) o la filtración de 10 ml. de muestra de orina por sujeto (para encontrar *S. haematobium*). Se extrajeron las cifras de positivos, falsos positivos, negativos y falsos negativos para las pruebas de antígenos y se realizaron metaanálisis mediante un modelo bivariante de regresión jerárquica.

**Resultados:**

Entre 1994 y 2014 se publicaron veintiséis estudios que cumplían los criterios de inclusión. Para detectar *S. mansoni*, una sola prueba de antígenos ofreció una sensibilidad combinada de 0,90 (intervalo de confianza, IC, del 95%: 0,84–0,94) y una especificidad combinada del 0,56 (IC del 95%: 0,39–0,71; *n* = 7) en comparación con una única prueba de Kato–Katz. Los valores correspondientes derivados de las comparaciones con entre dos y tres pruebas de Kato–Katz por sujeto fueron del 0,85% (IC del 95%: 0,80–0,88) y del 0,66 (IC del 95%: 0,53–0,76; *n* = 14), respectivamente. No parecía haber ventaja alguna a la hora de utilizar tres pruebas de antígenos por sujeto en lugar de una. En comparación con los resultados de la filtración de orina, las pruebas de antígenos de *S. haematobium* mostraron una sensibilidad y especificidad bajas. Los resultados de las pruebas de antígenos fueron mejores en zonas de alta endemicidad que en lugares de baja endemicidad.

**Conclusión:**

Las pruebas de antígenos pueden representar una herramienta eficaz para los programas de supervisión para el control de *S. mansoni*.

## ملخص

**الغرض:**

تقييم دقة الاختبارات التي تتم في أماكن الرعاية السريرية للمستضد المهبطي الدوراني في اكتشاف عدوى البلهارسيا.

**الطريقة:**

قمنا بإجراء بحث في قواعد معطيات MEDLINE، و EMBASE، و LILACS وغيرها من القواعد الببليوغرافية الأخرى للعثور على دراسات نشرت حتى 30 سبتمبر/أيلول 2015 والتي تم من خلالها وصف اختبار المستضد المهبطي الدوراني بالمقارنة مع واحد إلى ثلاثة اختبارات بتقنيةKato‑Katz لكل حالة – *لعدوى البلهارسيا المنسونية* – أو تقطير عينة واحدة بمقدار 10 مل من البول لكل حالة – *لعدوى البلهارسيا الدموية *. وقمنا باستخراج الأرقام المشيرة إلى النتائج الإيجابية الحقيقية، والنتائج الإيجابية الكاذبة، والنتائج السلبية الحقيقية، والنتائج السلبية الكاذبة لاختبار المستضد، وقمنا بإجراء تحليل تَلوي باستخدام نموذج التحوف التسلسلي المزدوج.

**النتائج:**

كانت هناك ست وعشرون دراسة منشورة في الفترة بين عامي 1994 و2014 ملائمة لمعايير الاختيار. في حالة اكتشاف *عدوى البلهارسيا المنسونية *، أظهر اختبار واحد للمستضد مقدارًا من الحساسية الجماعية يبلغ 0.90 (بنسبة أرجحية مقدارها 95‏%: 0.84–0.94) ونوعية جماعية بمقدار 0.56 (بنسبة أرجحية مقدارها ‏‏95% 0.71–0.39؛ *العدد * = 7) عندما تتم مقارنته مع اختبار واحد بتقنية Kato‑Katz. وكانت القيم المقابلة الناتجة عن المقارنات مع اثنين إلى ثلاثة اختبارات بتقنية Kato‑Katz لكل حالة تبلغ 0.85 (بنسبة أرجحية مقدارها 95‏%: 0.80–0.88) و0.66 (بنسبة أرجحية مقدارها 95‏%: 0.53–0.76؛ *العدد * = 14)، على التوالي. لقد بدا أنه لا توجد أية ميزة في استخدام ثلاثة اختبارات للمستضد لكل حالة بدلاً من استخدام اختبار واحد. لقد أظهر اختبار المستضد لعدوى *البلهارسيا الدموية * حساسية ضعيفة ونوعية ضعيفة عند مقارنته مع نتائج تقطير البول. وكان أداء اختبار المستضد أفضل في المناطق التي يوجد بها نسب توطين مرتفعة للعدوى أكثر من الأماكن التي يوجد بها نسب توطين منخفضة للعدوى.

**الاستنتاج:**

قد يمثل اختبار المستضد أداة فعالة لمراقبة البرامج المخصصة لمكافحة *عدوى البلهارسيا المنسونية *.

## 摘要

**目的:**

旨在评估血吸虫感染诊断中循环阴极抗原即时检测的准确性。

**方法:**

我们搜索了截止 2015 年 9 月 30 日前在美国联机医学文献分析和检索系统 (MEDLINE)、荷兰医学文摘数据库 (EMBASE)、拉丁美洲和加勒比健康科学文献库 (LILACS) 和其他书目数据库中发表的研究， 这些研究对每位研究对象的循环阴极抗原检测与一至三次加藤氏厚涂片检测进行对比描述——以检测*曼氏血吸虫*——或对每位研究对象的循环阴极抗原检测与一份 10ml 尿液样本过滤进行对比描述——以检测*埃及血吸虫*。 我们归纳整理了抗原检测中真阳性、假阳性、真阴性和假阴性结果的数量，并且利用多变量分层回归模型进行了元分析。

**结果:**

在 1994 年到 2014 年间发表的研究中，有二十六项符合纳入标准。 *曼氏血吸虫*检测中，与单次加藤氏厚涂片检测相比，单次抗原检测的汇总灵敏度为 0.90（95% 置信区间，CI: 0.84–0.94），且汇总特异度为 0.56（95% 置信区间: 0.39–0.71;*n* = 7）。 与每位研究对象的两至三次加藤氏厚涂片检测相比，相应数值分别为 0.85（95% 置信区间: 0.80-0.88）和 0.66（95% 置信区间: 0.53–0.76;*n* = 14）。 与一次抗原检测相比，对每位研究对象进行三次抗原检测后，并未发现明显优势。 当与尿液过滤相比，*埃及血吸虫*抗原检测表现出较差的灵敏度和特异度。 在高发地区，抗原检测的表现优于低发环境中的表现。

**结论:**

抗原检测可用作一种控制*曼氏血吸虫*的有效监测措施。

## Резюме

**Цель:**

Дать оценку точности тестирования на циркулирующий катодный антиген по месту лечения при диагностике шистосомоза.

**Методы:**

Был осуществлен поиск в базах данных MEDLINE, EMBASE, LILACS и других библиографических базах данных на предмет исследований, опубликованных до 30 сентября 2015 года, в которых результаты тестирования на циркулирующий катодный антиген сравнивались бы с результатами от 1 до 3 анализов проб, взятых у одного пациента, по методу Като-Кац для обнаружения Schistosoma mansoni или с результатами фильтрования одного образца мочи (10 мл), взятого у одного пациента, для обнаружения S. haematobium. Было подсчитано количество истинно положительных, ложно положительных, истинно отрицательных и ложно отрицательных результатов тестирования на антитела, и были проведены метаанализы с конструированием двумерной иерархической регрессии.

**Результаты:**

Двадцать шесть исследований, опубликованных в период между 1994 и 2014 годами, соответствовали критериям включения. При диагностике S. mansoni объединенная чувствительность метода при проведении одного теста на антитела составила 0,90 (95%-й доверительный интервал, ДИ: 0,84–0,94), а объединенная специфичность — 0,56 (95%-й ДИ: 0,39–0,71; n = 7) в сравнении с одним анализом по методу Като-Кац. Соответствующие значения, в сравнении с результатами от 2 до 3 анализов по методу Като-Кац для каждого пациента, были равны 0,85 (95%-й ДИ: 0,80–0,88) и 0,66 (95%-й ДИ: 0,53–0,76; n = 14) соответственно. Было установлено, что проведение трех тестов на антитела вместо одного не является целесообразным. В сравнении с результатами фильтрования мочи чувствительность и специфичность метода при тестировании на антитела для диагностики S. haematobium были неудовлетворительными. Эффективность тестирования на антитела была выше в зонах с высокой эндемичностью, чем в условиях с низкой эндемичностью.

**Вывод:**

Тестирование на антитела может послужить эффективным способом для программ мониторинга по борьбе с S. mansoni.

## Introduction

Schistosomiasis is common in low-income tropical and subtropical countries, especially where it is difficult to provide basic care at the peripheral level.^1^ Almost a billion people are estimated to be at risk of schistosome infection and over 200 million are infected.^2^^–^^5^ As there is a high risk of reinfection after treatment, repeated screening and treatment are important.^6^^,^^7^ *Schistosoma mansoni* and *S. japonicum* cause most cases of intestinal schistosomiasis while *S. haematobium* causes urogenital schistosomiasis.

Although the World Health Organization’s (WHO’s) strategy for schistosomiasis control was largely based on active case detection and treatment with praziquantel, mass treatment – with no prior diagnosis – is now increasingly employed in areas with high endemicity.^5^ Most diagnosis is based on Kato–Katz thick smears^8^ for intestinal schistosomiasis and urine filtration for urogenital schistosomiasis. The sensitivity of both of these diagnostic techniques depends on the severity of infection and often falls below 30% for mild infections.^9^^,^^10^ Although repeated sampling – e.g. the taking of several stool specimens on different days, from each subject, for Kato–Katz testing – can increase sensitivity, it also increases costs and the risk of false-positive results.

Since the introduction of mass drug administration within the preventive chemotherapy strategy, the prevalence and intensity of schistosome infection has fallen substantially in most settings and, in consequence, such infection has become harder to detect.^5^ Better but low-cost diagnostic tests are now needed to increase sensitivity without compromising specificity. It is possible to detect some schistosome infections by testing for either of two of the parasites’ secretory metabolites that have been linked with active infection: circulatory anodic antigen and circulatory cathodic antigen.^11^^–^^18^ A cassette assay for the point-of-care testing of urine samples for the latter antigen has been developed.^19^ When validated in settings in Africa, this assay was generally found to be much more sensitive – in the detection of *S. mansoni* infection – than the Kato–Katz test, although it appeared to suffer the same limitation when intensities of infection were low.^20^^–^^24^

Systematic reviews are widely regarded as providing the best evidence to inform health-care decisions.^25^^,^^26^ The systematic review and meta-analysis described below was commissioned by WHO to assess the diagnostic accuracy of point-of-care testing for circulatory cathodic antigen – hereafter called antigen testing. A Cochrane review was recently published on the same topic.^27^

The main aim of the review and meta-analysis was to evaluate the accuracy of antigen testing in the detection of all schistosome infections. We generally used the examination of two Kato–Katz thick smears of stools per subject as the reference standard in the detection of *S. mansoni* and *S. japonicum* and the filtration of 10 ml of urine per subject as the corresponding standard for *S. haematobium*.

## Methods

### Search methods

We searched MEDLINE, EMBASE and LILACS for relevant articles, in any language, recorded between the inception of each database and 30 September 2015. We also searched BIOSIS, Web of Science, Google Scholar, the Rapid Medical Diagnostics database, African Journals Online, Cochrane Infectious Diseases Group Specialized Register, the Cochrane Library 2015 and the metaRegister of Controlled Trials. We maximized the sensitivity of our search by using free texts based on the index test and target condition – i.e. antigen testing and schistosome infection, respectively. We also hand-checked the reference lists of relevant articles and textbooks and contacted experts in the field to see if they had any relevant but unpublished data.

### Inclusion criteria

We considered a study for inclusion if, for the detection of schistosome infection, it compared antigen testing with Kato–Katz tests and/or urine filtration, the pre-control infection status of the participants was not known, the same participants were checked using antigen tests and at least one reference test, and data on diagnostic accuracy were reported.

The data included in our review had to come from study participants whose stools had been checked for *S. mansoni* and/or *S. japonicum* eggs using the Kato–Katz test^8^ or whose urine had been checked for *S. haematobium* eggs using filtration of a 10 ml sample and microscopical examination of the filter.

### Diagnostic thresholds

Stool samples found to contain fewer than 100, 100–399 and more than 399 eggs per gram of faeces when examined as Kato–Katz smears were considered to come from participants with light, moderate and heavy infections, respectively. Urine that contained fewer than 51 or more than 50 eggs per 10-ml sample was considered to come from participants with light and heavy infections, respectively. All of the included results of antigen testing had been classifying qualitatively as: trace as negative, trace as positive or single, double or triple positive.

### Study selection

One author conducted the initial wide-ranging search of the literature. Two other authors then screened the results to identify those studies that were potentially relevant and useful. Full study reports were then obtained and checked to see if they satisfied several predefined inclusion criteria. Any discrepancies were resolved through discussion between the authors.

### Data extraction and management

Using a standardized form, two authors extracted study characteristics such as the country and year in which the study was conducted and the study design and the methods. Information on diagnostic criteria – e.g. the number of stool and urine samples examined per participant and the diagnostic thresholds employed – and epidemiological and demographic data – e.g. endemicity status, region where the study was conducted, participants’ prior treatment status, target population, sex, age and number of participants and whether diagnosis was delivered at the point of care – were also extracted.

We extracted the numbers of true positives, false positives, true negatives and false negatives for the antigen testing – using the results of a reference test as the gold standard. When necessary, we contacted the authors of the published articles on included studies to see if they could clarify or supplement the published results or provide raw data that we could use. If two or more communities were involved in a study, data were extracted for each community – with a link to the parent study.

### Data synthesis

Data were analysed and presented as sensitivities, specificities and false-positive rates, with their 95% confidence intervals (CIs). The meta-analyses were performed using the bivariate model specified by Reitsma et al.^28^ and the mada package in the R programming environment (R Foundation, Vienna, Austria).^29^ The model we used is equivalent to the hierarchical regression approach described by Rutter and Gatsonis.^30^^,^^31^ In the model, variance components are estimated by restricted maximum likelihood. To remove the need to adjust for confounders, we restricted our analyses to data from studies in which both index and reference standard tests were evaluated in the same participants. Subgroup effects were investigated by stratifying the analyses by age – categorized as preschool children and infants, school-aged children or adults – as well as the sensitivity of the reference standard and the background endemicity of either the intestinal schistosomiasis investigated – categorized as low, moderate or high – or the urinary schistosomiasis investigated – categorized as low or high.

### Heterogeneity and subgroup analysis

We assessed heterogeneity by inspecting forest plots for overlapping confidence intervals and outlying data. Although we generally considered a *P*-value below 0.05 to indicate statistical significance, we used a more sensitive threshold^32^^,^^33^ – i.e. a *P*-value below 0.10 – to indicate statistically significant heterogeneity. Where such significant heterogeneity was detected, we carried out subgroup analyses based on clinical and methodological differences.

We applied an exploratory analysis based on a latent class bivariate model^34^ to investigate the performance of antigen testing – compared with Kato–Katz tests used as the reference standard. For this analysis, Latent GOLD version 5.0 (Statistical Innovations Inc., Belmont, United States of America)^35^ was used to capture the between-study heterogeneity in sensitivity and specificity – assuming that our included studies belonged to one of several latent classes.^34^

## Results

**Fig. 1:**
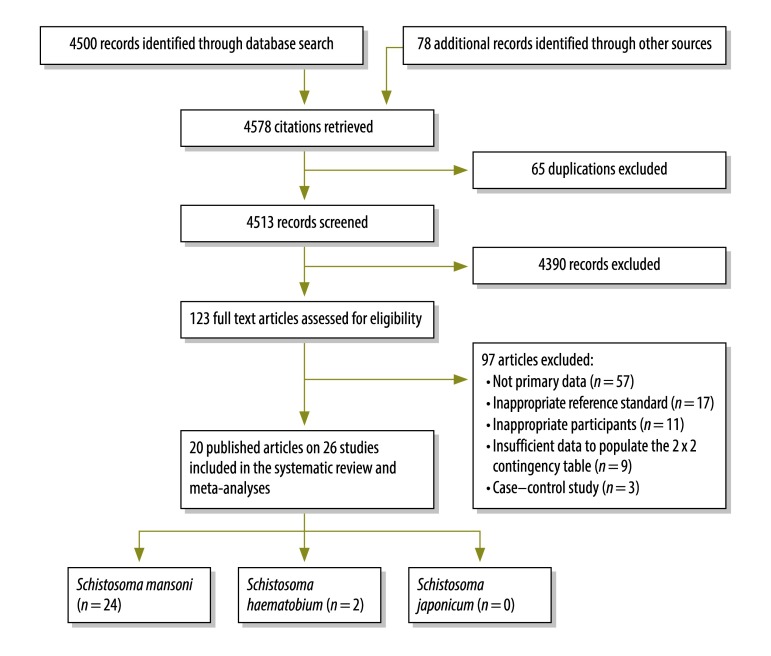
Selection of studies included in the systematic review and meta-analysis on the accuracy of point-of-care testing for circulatory cathodic antigen in the detection of schistosome infection

Note: Some reported articles were conducted in settings of low, moderate and high endemicity. We treated each of these articles as a report on three studies.

We retrieved 4578 records in the initial search. The data in 20 published articles on the 26 studies that met all of our inclusion criteria were included in the review (Fig. 1 and Table 1). In this article we present our main findings on the performance of antigen testing compared with Kato–Katz smears and urine filtration. More details on this topic and on other parts of the meta-analysis we conducted are available from the corresponding author.

**Table 1:** Characteristics of the studies included in the systematic review and meta-analysis on the accuracy of point-of-care testing for circulatory cathodic antigen in the detection of schistosome infection

Study	Country	Year	No. of study communities	Initial sample size	Participants	Ages (years)	Prevalence (%)**^a^**	CCA test investigated, and no. of samples per participant	No. of stool samples per participant**^b^**
Kremsner, 1994^17^	Cameroon	NR	1	148	Schoolchildren	4–13	NR	EIA (1)	1
De Clercq, 1997^36^	Mali	NR	2	NR^c^	Adults and children	NR	99.0	ELISA (1)	2
De Clercq, 1997^37^	Mali	1993	4	NR^d^	Adults and children	NR	NR	ELISA (1)	1
Legesse, 2007^38^	Ethiopia	2007	1	251	Adults and children	> 5	90.0 (schoolchildren)	Reagent strip (1)	1
Ayele, 2008^39^	Ethiopia	NR	1	206	Schoolchildren	4–21	47.6	Reagent strip (1)	NA
Legesse, 2008^40^	Ethiopia	2007	1	184	Schoolchildren	5–22	36.4	Reagent strip (1)	1
Midzi, 2009^41^	Zimbabwe	2006	1	265	Preschool children and schoolchildren	2–19	40.4	Reagent strip (1)	1
Stothard, 2009^42^	Uganda	2009	1	242	Infants and preschool children	< 6	> 50.0	Reagent strip (1)	2
Sousa-Figueiredo, 2010^43^	Uganda	2007 and 2009	NR	608	Preschool children and mothers	< 7^e^	16.0 and 43.3 (children), 29.2 and 60.0 (mothers) from either Lake Victoria or Lake Albert	Cassette (1)	2
Standley, 2010^44^	Kenya, United Republic of Tanzania	2009	11	171	Schoolchildren	6–17	68.6	Reagent strip (1)	1
Coulibaly, 2011^20^ (study 1)^f^	Côte d’Ivoire	2010	1	146	Children	8–12	32.9	Cassette (1, 2 or 3)	1, 2 or 3
Coulibaly, 2011^20^ (study 2)^f^	Côte d’Ivoire	2010	1	130	Children	8–12	53.1	Cassette (1, 2 or 3)	1, 2 or 3
Coulibaly, 2011^20^ (study 3)^f^	Côte d’Ivoire	2010	1	170	Children	8–12	91.8	Cassette (1, 2 or 3)	1, 2 or 3
Shane, 2011^45^	Kenya	2007	1	484	Children	1–15	38.8	Dipstick (1)	3
Tchuem Tchuenté, 2012^21^ (study 1)^f^	Cameroon	2010/2011	1	765	Schoolchildren	8–12	21.0	Cassette (1) and dipstick (NR)	3
Tchuem Tchuenté, 2012^21^ (study 2)^f^	Cameroon	2010/2011	1	765	Schoolchildren	8–12	41.8	Cassette (1) and dipstick (NR)	3
Tchuem Tchuenté, 2012^21^ (study 3)^f^	Cameroon	2010/2011	1	765	Schoolchildren	8–12	31.4	Cassette (1) and dipstick (NR)	3
Colley, 2013^22^	Cameroon, Côte d’Ivoire, Ethiopia, Kenya, Uganda	2010	5	4305	Schoolchildren	9–12	15.1 (Kenya), 25.0 (Uganda), 38.4 (Cameroon), 43.0 (Ethiopia) and 47.9 (Côte d’Ivoire)	Cassette (1)	1
Coulibaly, 2013^46^	Côte d’Ivoire	2011	2	242	Preschool children	< 6	23.1	Cassette (2)	2
Dawson, 2013^47^	Uganda	2011	NR	82	Preschool children	< 6	45.0	Cassette (1)	2
Erko, 2013^48^	Ethiopia	2010/2011	2	620	Schoolchildren	8–12	34.0	Cassette (1, 2 or 3)	1, 2 or 3
Koukounari, 2013^49^	Uganda	2005	1	446	Children and adults	7–16 and 17–76	NR	Cassette (1)	3
Sousa-Figueiredo, 2013^23^ (study 1)^ f^	Uganda	2009	NR	333	Preschool children	< 7	7.2	Dipstick (1)	1
Sousa-Figueiredo, 2013^23^ (study 2)^ f^	Uganda	2009	NR	337	Preschool children	< 7	16.9	Dipstick (1)	1
Sousa-Figueiredo, 2013^23^ (study 3)^ f^	Uganda	2009	NR	255	Preschool children	< 7	38.8	Dipstick (1)	1
Adriko, 2014^24^	Uganda	NR	5	500	Schoolchildren	7–13	8.0, 23.0 and 36.0 from low, moderate and high endemic areas, respectively	Cassette (1)	1, 2 or 3

CCA: circulatory cathodic antigen; EIA: enzyme immunoassay; ELISA: enzyme-linked immunosorbent assay; NA: not applicable; NR: not reported.

^a^ Of infection with the schistosome species of interest, as indicated by the results of the reference test.

^b^ Each examined as a duplicate Kato–Katz smear.

^c^ Overall, 352 serum, 134 stool and 337 urine samples were investigated

^d^ Overall, 348 blood, 324 stool and 431 urine samples were investigated.

^e ^Reported age of children.

^f^ The article was conducted in settings of low, moderate and high endemicity. We treated the publication as a report on three studies, which we designated studies 1, 2 and 3.

**Fig. 2:**
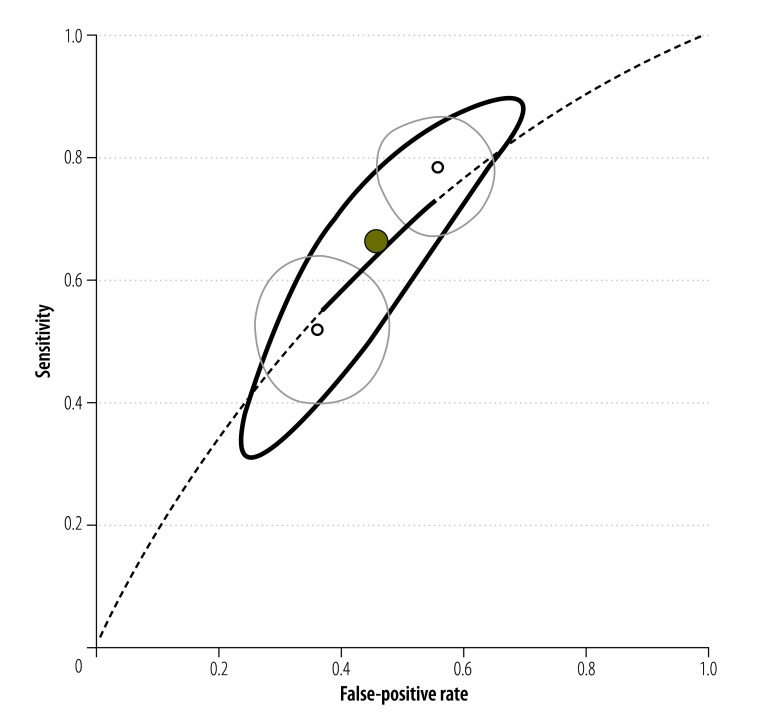
Single point-of-care testing for circulatory cathodic antigen in the detection of *Schistosoma haematobium* infection: *summary receiver-operating characteristic* curve

All of the included studies were conducted in Africa – i.e. in East Africa,^23^^,^^24^^,^^38^^–^^40^^,^^42^^–^^45^^,^^47^^–^^49^ West Africa^17^^,^^20^^,^^21^^,^^36^^,^^37^^,^^46^ southern Africa^41^ or five countries scattered across Africa.^22^ Most were cross-sectional and none was a randomized control trial. Three of the studies were conducted in the 1990s and used the older version of the test for circulatory cathodic antigen.^17^^,^^36^^,^^37^ The rest were conducted after 2000. All but two of the included studies involved the detection of *S. mansoni*. Two involved the detection of *S. haematobium* (Fig. 2) and none investigated *S. japonicum* infections.

AUC: area under the curve.

Notes: The analysed data came from two studies^39^^,^^41^ in which, for each participant, filtration of a 10-ml urine sample served as the reference standard. Each participant was tested once for antigen, using reagent strips. In the antigen testing, all positive results – including trace positives – were considered indicative of infection. The graph contains six separate types of information, represented by six separate types of graphical feature. Hollow circles represent the point estimates for the sensitivity and specificity of each study. Each of these circles is surrounded by a light grey oval, which presents the 95% credible region associated with that particular study. Similarly, the summary models – produced by pooling the estimates from each of the studies using a standard bivariate model – are presented both as a point estimate, represented by a solid green circle, and an associated 95% credible region, represented by the black oval. In addition to this, the best estimate for how the sensitivity and specificity vary with the diagnostic threshold adopted is represented by a line which runs from the bottom left to the top right portion of the graph. The solid section of this line represents interpolated estimates – which fill in the gaps between the studies available – whereas the dashed parts of this line are extrapolated from the data and are therefore more dependent on the modelling assumptions. Both the interpolated and the extrapolated parts of this line are needed to estimate the AUC, which is a measure of the antigen test’s diagnostic accuracy.

Each of two publications^20^^,^^21^ reported studies conducted in settings of low, moderate and high endemicity. We treated each of these publications as a report on three studies, which we designated studies 1, 2 and 3. As another investigation^49^ had both adult and child participants and reported data separately for these two age groups, we were able to analyse its data as if they came from two studies. Since one publication^22^ included some data from primary research represented by other articles included in our analysis, we had to be careful to avoid duplicate analyses. When contacted, the authors of three included articles^23^^,^^24^^,^^44^ provided useful unpublished data.

### One antigen test per participant

#### Versus single Kato–Katz

**Fig. 3:**
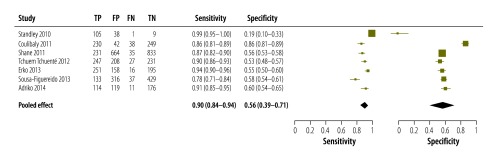
Accuracy of single point-of-care testing for circulatory cathodic antigen in the detection of *Schistosoma mansoni* infection

FN: false negatives; FP: false positives; TN: true negatives; TP: true positives.

Notes: The analysed data came from seven studies^20^^,^^21^^,^^23^^,^^24^^,^^44^^,^^45^^,^^48^ in which, for each participant, examination of duplicate Kato–Katz smears of a single stool sample served as the reference standard. In the antigen testing, all positive results – including trace positives – were considered indicative of infection. Data points for two studies^21^^,^^24^ were extracted from a report^22^ that included primary data from a multi-country study in Africa. Although reagent strips were used as the antigen tests in two of the studies,^44^^,^^45^ most of the data came from studies in which cassette assays had been used.

**Fig. 4:**
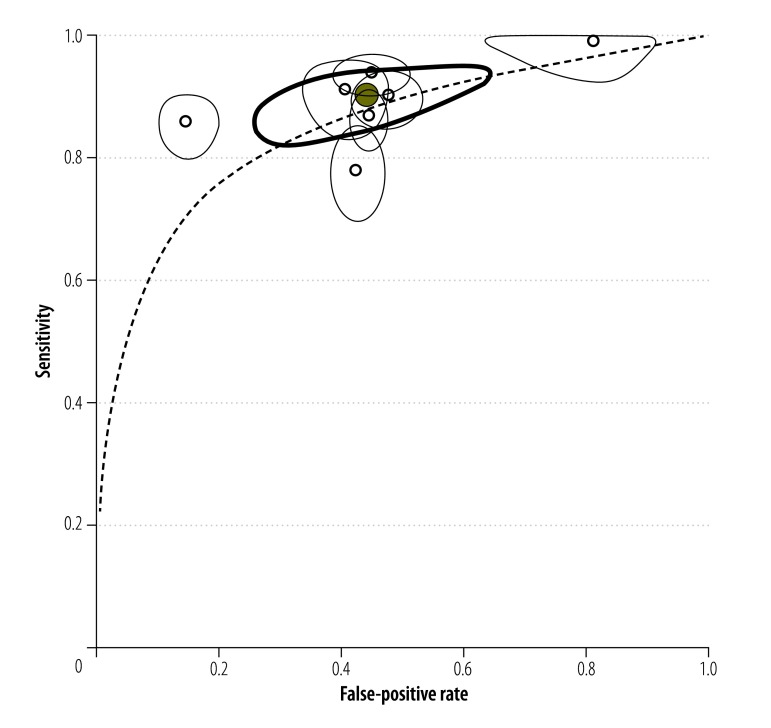
Single point-of-care testing for circulatory cathodic antigen in the detection of *Schistosoma mansoni* infection: *summary receiver-operating characteristic* curve

The accuracy of single antigen testing compared with single Kato–Katz reference testing – i.e. the examination of two smears of a single stool sample per participant – for the detection of *S. mansoni* infection had been investigated in seven studies,^21^^,^^20^^,^^48^^,^^44^^,^^45^^,^^23^^,^^24^ in Cameroon, Côte d’Ivoire, Ethiopia, Kenya and Uganda. Our meta-analysis of the data from these studies indicated that the antigen test had a high pooled sensitivity (0.90; 95% CI: 0.84–0.94) but a low pooled specificity (0.56; 95% CI: 0.39–0.71; Fig. 3). The area under the corresponding receiver-operating characteristic curve indicated that the antigen test had an accuracy of 0.86 (Fig. 4; available at: http://www.who.int/bulletin/volumes/94/7/15-158741). The same curve indicated that there had been wide variation in the antigen test’s false-positive rate when the test had been used to detect *S. mansoni* infection.

AUC: area under the curve.

Notes: The analysed data came from seven studies^20^^,^^21^^,^^23^^,^^24^^,^^44^^,^^45^^,^^48^ in which, for each participant, examination of duplicate Kato–Katz smears of a single stool sample served as the reference standard. In the antigen testing, all positive results – including trace positives – were considered indicative of infection. Data points for two studies^21^^,^^24^ were extracted from a report^22^ that included primary data from a multi-country study in Africa. Although reagent strips were used as the antigen tests in two of the studies,^44^^,^^45^ most of the data came from studies in which cassette assays had been used. The graph contains six separate types of information, represented by six separate types of graphical feature. Hollow circles represent the point estimates for the sensitivity and specificity of each study. Each of these circles is surrounded by a light grey oval, which presents the 95% credible region associated with that particular study. Similarly, the summary models – produced by pooling the estimates from each of the studies using a standard bivariate model – are presented both as a point estimate, represented by a solid green circle, and an associated 95% credible region, represented by the black oval. In addition to this, the best estimate for how the sensitivity and specificity vary with the diagnostic threshold adopted is represented by a line which runs from the bottom left to the top right portion of the graph. The solid section of this line represents interpolated estimates – which fill in the gaps between the studies available – whereas the dashed parts of this line are extrapolated from the data and are therefore more dependent on the modelling assumptions. Both the interpolated and the extrapolated parts of this line are needed to estimate the AUC, which is a measure of the antigen test’s diagnostic accuracy.

#### Versus triple Kato–Katz

**Fig. 5:**
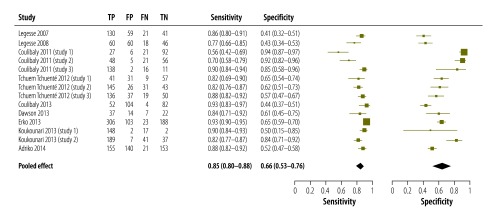
Accuracy of single point-of-care testing for circulatory cathodic antigen in the detection of *Schistosoma mansoni* infection

FN: false negatives; FP: false positives; TN: true negatives; TP: true positives.

Notes: The analysed data came from 14 studies^20^^,^^21^^,^^24^^,^^32^^,^^38^^,^^40^^,^^46^^,^^48^^,^^49^ in which, for each participant, examination of duplicate Kato–Katz smears of three or, in one study,^32^ two consecutive stool samples – in two studies,^38^^,^^40^ combined with the results of formol–ether concentration – served as the reference standard. In the antigen testing, all positive results – including trace positives – were considered indicative of infection. Although reagent strips were used as the antigen tests in two of the studies,^38^^,^^40^ most of the data came from studies in which cassette assays had been used. The data in one report^49^ that presented separate results for children aged 7–16 years and adults older than 16 years were treated as if they came from two independent studies.

In 14 studies on the detection of *S. mansoni* infection – described in nine articles^20^^,^^21^^,^^24^^,^^32^^,^^38^^,^^40^^,^^46^^,^^48^^,^^49^ – single antigen testing had been compared with triple Kato–Katz reference testing – i.e. the examination of two smears of each of three consecutive stool samples per participant. When pooled, these comparisons indicated that the antigen test had a sensitivity of 0.85 (95% CI: 0.80–0.88) and a specificity of 0.66 (95% CI: 0.53–0.76). The wide CIs of some of the studies indicated the effects of small sample sizes. While the estimates of the antigen test’s sensitivity showed some consistency, there was huge variation in the corresponding estimates of specificity (Fig. 5).

#### Versus combined antigen test and Kato–Katz

One of the studies we included in our analysis^24^ had used the combined results of single antigen testing with single Kato–Katz reference testing in evaluating the performance of the antigen test when detecting *S. mansoni* infection. In this study, single antigen testing had been found to have a high sensitivity (90%) and optimal specificity (100%).

### Three antigen tests per participant

#### Versus combined antigen test and Kato–Katz

In the study just described,^24^ the use of three antigen tests per participant led to slightly higher sensitivity (96%) and left specificity unchanged (100%).

#### Versus triple Kato–Katz

**Fig. 6:**
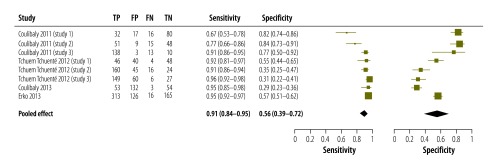
Accuracy of double or triple point-of-care testing for circulatory cathodic antigen in the detection of *Schistosoma mansoni* infection

FN: false negatives; FP: false positives; TN: true negatives; TP: true positives.

Notes: The analysed data came from eight studies^20^^,^^21^^,^^24^^,^^46^ in which, for each participant, examination of duplicate Kato–Katz smears of three – or, in one study,^46^ two – consecutive stool samples served as the reference standard. Each participant was tested three times – or, in one study,^46^ twice – for antigen, using cassette assays. In the antigen testing, all positive results – including trace positives – were considered indicative of infection.

In eight of the studies we included in our analysis – i.e. three from Cameroon,^21^ four from Côte d’Ivoire^20^^,^^46^ and one from Ethiopia^48^ – triple antigen testing for the detection of *S. mansoni* infection was compared with triple Kato–Katz reference testing. The meta-analysis of the data from these studies showed that triple antigen testing gave a pooled sensitivity of 0.91 (95% CI: 0.84–0.95) and a pooled specificity of 0.56 (95% CI: 0.39–0.72) (Fig. 6). Although the sensitivities of the triple antigen testing appeared to be fairly consistent across the studies, the corresponding specificities showed wide CIs and much between-study variability.

### Latent class analysis

We analysed 32 data points from studies included in this review and identified two latent classes for the antigen testing (Table 2). 

**Table 2:** Latent class analysis of the studies on the accuracy of point-of-care testing for circulatory cathodic antigen in the detection of schistosome infection included in the meta-analysis

Latent class and study	CCA test investigated, and no. of samples per participant	No. of stool samples per participant**^a^**
**Latent class 1**		
Coulibaly, 2011	Cassette (1)	1
Coulibaly, 2011 (study 1)	Cassette (1)	3
Coulibaly, 2011 (study 1)	Cassette (3)	3
Coulibaly, 2011 (study 2)	Cassette (1)	3
Coulibaly, 2011 (study 2)	Cassette (3)	3
Coulibaly, 2011 (study 3)	Cassette (1)	3
Koukounari, 2013 (study 2)	Cassette (1)	3
**Latent class 2**		
Legesse, 2007	Cassette (1)	1^b^
Legesse, 2008	Reagent strip (1)	1^b^
Standley, 2010	Cassette (1)	1
Shane, 2011	Cassette (1)	1
Coulibaly, 2011 (study 3)	Cassette (3)	3
Tchuem Tchuenté, 2012	Cassette (1)	1
Tchuem Tchuenté, 2012 (study 1)	Cassette (1)	3
Tchuem Tchuenté, 2012 (study 1)	Cassette (3)	3
Tchuem Tchuenté, 2012 (study 2)	Cassette (1)	3
Tchuem Tchuenté, 2012 (study 2)	Cassette (3)	3
Tchuem Tchuenté, 2012 (study 3)	Cassette (1)	3
Tchuem Tchuenté, 2012 (study 3)	Cassette (3)	3
Coulibaly, 2013	Cassette (1)	3
Coulibaly, 2013	Cassette (2)	2
Dawson, 2013	Cassette (1)	2
Erko, 2013	Cassette (1)	1
Erko, 2013	Cassette (1)	3
Erko, 2013	Cassette (3)	3
Koukounari, 2013 (study 1)	Cassette (1)	3
Sousa-Figueiredo, 2013	Cassette (1)	1
Sousa-Figueiredo, 2013 (study 1)	Cassette (1)	1
Sousa-Figueiredo, 2013 (study 2)	Cassette (1)	1
Sousa-Figueiredo, 2013 (study 3)	Cassette (1)	1
Adriko, 2014	Cassette (1)	1
Adriko, 2014	Cassette (1)	3

CCA: circulatory cathodic antigen.

^a^ Each examined as a duplicate Kato–Katz smear.

^b^ Stool samples were also checked by formol–ether concentration.

## Discussion

In this review, we were disappointed by the lack of a relevant randomized controlled trial. Most of the data we analysed came from cross-sectional studies. Despite the variability in the design of the studies we investigated, including variation in the format of the antigen tests employed, the studies gave fairly consistent results. An independent study found no batch-to-batch variation in the cassette version of the antigen test we investigated, negligible intra-reader variability (2%) and substantial agreement in the inter-reader reliability of the test.^50^

As all the studies we included in our analysis were conducted in Africa and most only assessed the performance of antigen testing for detecting *S. mansoni* infection, there needs to be much caution in generalizing our findings to other endemic areas and other schistosome species. Additional studies – on the detection of *S. mansoni* beyond Africa and on the detection of other schistosome species throughout the tropics and subtropics – are encouraged.^51^

The finding that the antigen test performed better when endemicity was high than when it was low has both practice and control implications. As schistosome control becomes more successful, antigen testing may have no advantage over Kato–Katz smears or urine filtration. As there is no test for schistosome infection that has 100% sensitivity and 100% specificity, the apparent performance of any index test is partially dependent on the performance and other characteristics of the reference test or tests. Microscopy performed on multiple stool or urine samples – as appropriate – might be considered to be an effective parasitological gold standard.^52^ Researchers have suggested that a useful gold standard might be created by combining the results of the index and reference tests.^52^ However, the combined results of antigen and Kato–Katz testing might be adversely affected by false-positive antigen tests, false-negative Kato–Katz tests and interdependence in the two sets of results. When investigating the performance of antigen testing, it may be better to use a test with a low false-positive rate as the reference for sensitivity – e.g. Kato–Katz testing of multiple stool samples collected on different days from each participant – and to evaluate the test’s specificity using participants from non-endemic areas. An alternative approach would be to use a predicted gold standard at population level – like the pocket chart described by researchers.^53^ Although the combined results of antigen and Kato–Katz testing are not being employed in any current control programme, they may become a diagnostic option in the future.

The absence of a clear and accurate reference standard creates additional uncertainty in the meta-analysis of results data from any diagnostic test. After investigating heterogeneity patterns through latent class bivariate analysis,^34^ we identified two latent classes (Table 2). As the substantial variation we observed in the diagnostic accuracy of the antigen test could not be entirely explained by a threshold effect, we conducted subgroup analyses. The results indicated that the number of urine samples tested per participant had little effect on the antigen test’s sensitivity and specificity (data available from corresponding author). When we attempted to relate latent class to several background factors, we found that the number of urine samples tested per participant and the study year and country had little effect on the antigen test’s accuracy (data available from corresponding author). Several other factors that could not be thoroughly explored at this stage – e.g. age, endemicity and effect of treatment – require further investigation.

If not fully cured, most individuals covered by mass administrations of praziquantel will have light infections that can easily be missed by insensitive tests. Although we made no comparison of the antigen test’s performance before and after treatment, we evaluated the effect of endemicity on the performance of antigen testing with the specific aim of determining how the test would perform in settings with generally low intensities of infection. We appreciate the fact that important additional evidence could have come from post-treatment studies and – given that our meta-analysis involved mostly cross-sectional studies – there may have been unknown confounding factors. We are also aware that our analysis was limited to data from Africa recorded in 20 articles. Despite these limitations, the findings of the studies included in our analysis seem fairly consistent. Although the quality of the included studies was not formally assessed, potential sources of heterogeneity were explored. Our main conclusions are consistent with the available evidence shown and are likely to be reliable.

In conclusion, the antigen testing we evaluated appears to represent an effective, easy and low-cost tool for mapping and monitoring programmes for the control of *S. mansoni* and, possibly, *S. haematobium*. Well-designed studies involving head-to-head comparisons of the cost and cost–effectiveness of antigen testing and either Kato–Katz smears or urine filtration and evaluations of the performance of antigen testing post-treatment are recommended.

## References

[1] Chitsulo L, Engels D, Montresor A, Savioli L. The global status of schistosomiasis and its control. Acta Trop. 2000 10 23;77(1):41–51. 10.1016/S0001-706X(00)00122-4 10996119 PMC5633072

[2] Engels D, Chitsulo L, Montresor A, Savioli L. The global epidemiological situation of schistosomiasis and new approaches to control and research. Acta Trop. 2002 5;82(2):139–46. 10.1016/S0001-706X(02)00045-1 12020886 PMC5633073

[3] Hotez PJ, Molyneux DH, Fenwick A, Ottesen E, Sachs SE, Sachs JD. Incorporating a rapid-impact package for neglected tropical diseases with programs for HIV/AIDS, tuberculosis, and malaria. PLoS Med. 2006;3(5):e102. 10.1371/journal.pmed.0040277 16435908 PMC1351920

[4] Steinmann P, Keiser J, Bos R, Tanner M, Utzinger J. Schistosomiasis and water resources development: systematic review, meta-analysis, and estimates of people at risk. Lancet Infect Dis. 2006 7;6(7):411–25. 10.1016/S1473-3099(06)70521-7 16790382

[5] Schistosomiasis. WHO progress report 2001–2011 and strategic plan 2012–2020. Geneva: World Health Organization; 2012.

[6] Prevention and control of schistosomiasis and soil-transmitted helminthiasis. [Technical Report Series No. 912]. Geneva: World Health Organization; 2002. 12592987

[7] King CH, Dangerfield-Cha M. The unacknowledged impact of chronic schistosomiasis. Chronic Illn. 2008 3;4(1):65–79. 10.1177/1742395307084407 18322031

[8] Katz N, Chaves A, Pellegrino J. A simple device for quantitative stool thick-smear technique in schistosomiasis mansoni. Rev Inst Med Trop Sao Paulo. 1972 Nov-Dec;14(6):397–400. 4675644

[9] Booth M, Vounatsou P, N’goran EK, Tanner M, Utzinger J. The influence of sampling effort and the performance of the Kato-Katz technique in diagnosing Schistosoma mansoni and hookworm co-infections in rural Côte d’Ivoire. Parasitology. 2003 12;127(6):525–31. 10.1017/S0031182003004128 14700188

[10] Raso G, Vounatsou P, McManus DP, N’Goran EK, Utzinger J. A Bayesian approach to estimate the age-specific prevalence of Schistosoma mansoni and implications for schistosomiasis control. Int J Parasitol. 2007 11;37(13):1491–500. 10.1016/j.ijpara.2007.05.004 17583713 PMC2756495

[11] Berggren WL, Weller TH. Immunoelectrophoretic demonstration of specific circulating antigen in animals infected with Schistosoma mansoni. Am J Trop Med Hyg. 1967 9;16(5):606–12. 6053529 10.4269/ajtmh.1967.16.606

[12] Gold R, Rosen FS, Weller TH. A specific circulating antigen in hamsters infected with Schistosoma mansoni. Detection of antigen in serum and urine, and correlation between antigenic concentration and worm burden. Am J Trop Med Hyg. 1969 7;18(4):545–52. 5795447 10.4269/ajtmh.1969.18.545

[13] Deelder AM, van Dam GJ, Kornelis D, Fillié YE, van Zeyl RJ. Schistosoma: analysis of monoclonal antibodies reactive with the circulating antigens CAA and CCA. Parasitology. 1996 1;112(1):21–35. 10.1017/S0031182000065045 8587799

[14] Kelly C. Molecular studies of schistosome immunity. In: Rollinson D, Simpson AJG, editors. The biology of schistosomes; from genes to latrines. London: Academic Press; 1987 pp. 265–93.

[15] De Jonge N, Gryseels B, Hilberath GW, Polderman AM, Deelder AM. Detection of circulating anodic antigen by ELISA for seroepidemiology of schistosomiasis mansoni. Trans R Soc Trop Med Hyg. 1988;82(4):591–4. 10.1016/0035-9203(88)90523-8 3151417

[16] De Jonge N, Fillié YE, Hilberath GW, Krijger FW, Lengeler C, de Savigny DH, et al. Presence of the schistosome circulating anodic antigen (CAA) in urine of patients with Schistosoma mansoni or S. haematobium infections. Am J Trop Med Hyg. 1989 11;41(5):563–9. 2510528 10.4269/ajtmh.1989.41.563

[17] Kremsner PG, Enyong P, Krijger FW, De Jonge N, Zotter GM, Thalhammer F, et al. Circulating anodic and cathodic antigen in serum and urine from Schistosoma haematobium-infected Cameroonian children receiving praziquantel: a longitudinal study. Clin Infect Dis. 1994 3;18(3):408–13. 10.1093/clinids/18.3.408 8011824

[18] van Lieshout L, Polderman AM, Deelder AM. Immunodiagnosis of schistosomiasis by determination of the circulating antigens CAA and CCA, in particular in individuals with recent or light infections. Acta Trop. 2000 10 23;77(1):69–80. 10.1016/S0001-706X(00)00115-7 10996122

[19] van Dam GJ, Wichers JH, Ferreira TM, Ghati D, van Amerongen A, Deelder AM. Diagnosis of schistosomiasis by reagent strip test for detection of circulating cathodic antigen. J Clin Microbiol. 2004 12;42(12):5458–61. 10.1128/JCM.42.12.5458-5461.2004 15583265 PMC535219

[20] Coulibaly JT, Knopp S, N’Guessan NA, Silue KD, Furst T, Lohourignon LK, et al. Accuracy of urine circulating cathodic antigen (CCA) test for S. mansoni diagnosis in different settings of Côte d’Ivoire. PLoS Negl Trop Dis. 2011;5(11):e1384. 10.1371/journal.pntd.0001384 22132246 PMC3222626

[21] Tchuem Tchuenté L-A, Kueté Fouodo CJ, Kamwa Ngassam RI, Sumo L, Dongmo Noumedem C, Kenfack CM, et al. Evaluation of circulating cathodic antigen (CCA) urine-tests for diagnosis of Schistosoma mansoni infection in Cameroon. PLoS Negl Trop Dis. 2012;6(7):e1758. 10.1371/journal.pntd.0001758 22860148 PMC3409114

[22] Colley DG, Binder S, Campbell C, King CH, Tchuem Tchuenté LA, N’Goran EK, et al. A five-country evaluation of a point-of-care circulating cathodic antigen urine assay for the prevalence of Schistosoma mansoni. Am J Trop Med Hyg. 2013 3;88(3):426–32. 10.4269/ajtmh.12-0639 23339198 PMC3592520

[23] Sousa-Figueiredo JC, Betson M, Kabatereine NB, Stothard JR. The urine circulating cathodic antigen (CCA) dipstick: a valid substitute for microscopy for mapping and point-of-care diagnosis of intestinal schistosomiasis. PLoS Negl Trop Dis. 2013;7(1):e2008. 10.1371/journal.pntd.0002008 23359826 PMC3554525

[24] Adriko M, Standley CJ, Tinkitina B, Tukahebwa EM, Fenwick A, Fleming FM, et al. Evaluation of circulating cathodic antigen (CCA) urine-cassette assay as a survey tool for Schistosoma mansoni in different transmission settings within Bugiri District, Uganda. Acta Trop. 2014 8;136:50–7. 10.1016/j.actatropica.2014.04.001 24727052

[25] Egger M, Smith GD. Meta-analysis. Potentials and promise. BMJ. 1997 11 22;315(7119):1371–4. 10.1136/bmj.315.7119.1371 9432250 PMC2127866

[26] Higgins JPT, Green S, editors. Cochrane handbook for systematic reviews of interventions. Version 5.1.0. Oxford: The Cochrane Collaboration; 2011.

[27] Ochodo EA, Gopalakrishna G, Spek B, Reitsma JB, van Lieshout L, Polman K, et al. Circulating antigen tests and urine reagent strips for diagnosis of active schistosomiasis in endemic areas. Cochrane Database Syst Rev. 2015;3:CD009579. 10.1002/14651858.CD009579.pub2 25758180 PMC4455231

[28] Reitsma JB, Glas AS, Rutjes AW, Scholten RJ, Bossuyt PM, Zwinderman AH. Bivariate analysis of sensitivity and specificity produces informative summary measures in diagnostic reviews. J Clin Epidemiol. 2005 10;58(10):982–90. 10.1016/j.jclinepi.2005.02.022 16168343

[29] Doebler P. mada: meta-analysis of diagnostic accuracy. R package version 0.5.7. Vienna: University of Economics and Business; 2015. Available from: http://cran.r-project.org/web/packages/mada/index.html [cited 2016 Feb 25].

[30] Harbord RM, Deeks JJ, Egger M, Whiting P, Sterne JA. A unification of models for meta-analysis of diagnostic accuracy studies. Biostatistics. 2007 4;8(2):239–51. 10.1093/biostatistics/kxl004 16698768

[31] Rutter CM, Gatsonis CA. A hierarchical regression approach to meta-analysis of diagnostic test accuracy evaluations. Stat Med. 2001 10 15;20(19):2865–84. 10.1002/sim.942 11568945

[32] DerSimonian R, Laird N. Meta-analysis in clinical trials. Control Clin Trials. 1986 9;7(3):177–88. 10.1016/0197-2456(86)90046-2 3802833

[33] Bossuyt P, Davenport C, Deeks J, Hyde C, Leeflang M, Scholten R. Chapter 11: Interpreting results and drawing conclusions. In: Deeks JJ, Bossuyt PM, Gatsonis C, editors. Cochrane handbook for systematic reviews of diagnostic test accuracy, Version 0.9. Oxford: The Cochrane Collaboration; 2013. Available from: http://methods.cochrane.org/sdt/sites/methods.cochrane.org.sdt/files/uploads/DTA%20Handbook%20Chapter%2011%20201312.pdf [cited 2013 Dec 13].

[34] Eusebi P, Reitsma JB, Vermunt JK. Latent class bivariate model for the meta-analysis of diagnostic test accuracy studies. BMC Med Res Methodol. 2014;14(1):88. 10.1186/1471-2288-14-88 25015209 PMC4105799

[35] Vermunt JK, Magidson J. Technical guide for latent GOLD 5.0: basic, advanced, and syntax. Belmont: Statistical Innovations Inc.; 2013. Available from: http://www.statisticalinnovations.com/latent-gold-5-1/ [cited 2016 Feb 25].

[36] De Clercq D, Sacko M, Vercruysse J, vanden Bussche V, Landouré A, Diarra A, et al. Assessment of cure by detection of circulating antigens in serum and urine, following schistosomiasis mass treatment in two villages of the Office du Niger, Mali. Acta Trop. 1997 12;68(3):339–46. 10.1016/S0001-706X(97)00111-3 9492918

[37] De Clercq D, Sacko M, Vercruysse J, vanden Bussche V, Landouré A, Diarra A, et al. Circulating anodic and cathodic antigen in serum and urine of mixed Schistosoma haematobium and S. mansoni infections in Office du Niger, Mali. Trop Med Int Health. 1997 7;2(7):680–5. 10.1046/j.1365-3156.1997.d01-354.x 9270735

[38] Legesse M, Erko B. Field-based evaluation of a reagent strip test for diagnosis of Schistosoma mansoni by detecting circulating cathodic antigen in urine before and after chemotherapy. Trans R Soc Trop Med Hyg. 2007 7;101(7):668–73. 10.1016/j.trstmh.2006.11.009 17368699

[39] Ayele B, Erko B, Legesse M, Hailu A, Medhin G. Evaluation of circulating cathodic antigen (CCA) strip for diagnosis of urinary schistosomiasis in Hassoba school children, Afar, Ethiopia. Parasite. 2008 3;15(1):69–75. 10.1051/parasite/2008151069 18416249

[40] Legesse M, Erko B. Field-based evaluation of a reagent strip test for diagnosis of schistosomiasis mansoni by detecting circulating cathodic antigen (CCA) in urine in low endemic area in Ethiopia. Parasite. 2008 6;15(2):151–5. 10.1051/parasite/2008152151 18642508

[41] Midzi N, Butterworth AE, Mduluza T, Munyati S, Deelder AM, van Dam GJ. Use of circulating cathodic antigen strips for the diagnosis of urinary schistosomiasis. Trans R Soc Trop Med Hyg. 2009 1;103(1):45–51. 10.1016/j.trstmh.2008.08.018 18951599

[42] Stothard JR. Improving control of African schistosomiasis: towards effective use of rapid diagnostic tests within an appropriate disease surveillance model. Trans R Soc Trop Med Hyg. 2009 4;103(4):325–32. 10.1016/j.trstmh.2008.12.012 19171359

[43] Sousa-Figueiredo JC, Pleasant J, Day M, Betson M, Rollinson D, Montresor A, et al. Treatment of intestinal schistosomiasis in Ugandan preschool children: best diagnosis, treatment efficacy and side-effects, and an extended praziquantel dosing pole. In Health. 2010 6;2(2):103–13. 10.1016/j.inhe.2010.02.003 20640034 PMC2892744

[44] Standley CJ, Lwambo NJS, Lange CN, Kariuki HC, Adriko M, Stothard JR. Performance of circulating cathodic antigen (CCA) urine-dipsticks for rapid detection of intestinal schistosomiasis in schoolchildren from shoreline communities of Lake Victoria. Parasit Vectors. 2010;3(1):7. 10.1186/1756-3305-3-7 20181101 PMC2828997

[45] Shane HL, Verani JR, Abudho B, Montgomery SP, Blackstock AJ, Mwinzi PN, et al. Evaluation of urine CCA assays for detection of Schistosoma mansoni infection in Western Kenya. PLoS Negl Trop Dis. 2011;5(1):e951. 10.1371/journal.pntd.0000951 21283613 PMC3026766

[46] Coulibaly JT, N’Gbesso YK, Knopp S, N’Guessan NA, Silué KD, van Dam GJ, et al. Accuracy of urine circulating cathodic antigen test for the diagnosis of Schistosoma mansoni in preschool-aged children before and after treatment. PLoS Negl Trop Dis. 2013;7(3):e2109. 10.1371/journal.pntd.0002109 23556011 PMC3605147

[47] Dawson EM, Sousa-Figueiredo JC, Kabatereine NB, Doenhoff MJ, Stothard JR. Intestinal schistosomiasis in preschool-aged children of Lake Albert, Uganda: diagnostic accuracy of a rapid test for detection of anti-schistosome antibodies. Trans R Soc Trop Med Hyg. 2013 10;107(10):639–47. 10.1093/trstmh/trt077 23976783

[48] Erko B, Medhin G, Teklehaymanot T, Degarege A, Legesse M. Evaluation of urine-circulating cathodic antigen (Urine-CCA) cassette test for the detection of Schistosoma mansoni infection in areas of moderate prevalence in Ethiopia. Trop Med Int Health. 2013 8;18(8):1029–35. 10.1111/tmi.12117 23590255

[49] Koukounari A, Donnelly CA, Moustaki I, Tukahebwa EM, Kabatereine NB, Wilson S, et al. A latent Markov modelling approach to the evaluation of circulating cathodic antigen strips for schistosomiasis diagnosis pre- and post-praziquantel treatment in Uganda. PLOS Comput Biol. 2013;9(12):e1003402. 10.1371/journal.pcbi.1003402 24367250 PMC3868541

[50] Mwinzi PN, Kittur N, Ochola E, Cooper PJ, Campbell CH Jr, King CH, et al. Additional evaluation of the point-of-contact circulating cathodic antigen assay for Schistosoma mansoni infection. Front Public Health. 2015;3:48. 10.3389/fpubh.2015.00048 25853117 PMC4365547

[51] van Dam GJ, Odermatt P, Acosta L, Bergquist R, de Dood CJ, Kornelis D, et al. Evaluation of banked urine samples for the detection of circulating anodic and cathodic antigens in Schistosoma mekongi and S. japonicum infections: a proof-of-concept study. Acta Trop. 2015 1;141 Pt B:198–203. 10.1016/j.actatropica.2014.09.003 25225158

[52] Deelder AM, van Dam GJ, van Lieshout L. Response to: accuracy of circulating cathodic antigen tests for rapid mapping of Schistosoma mansoni and S. haematobium infections in Southern Sudan by RA Ashton et al. (2011) Trop Med Int Health 16, pp. 1099-1103. Trop Med Int Health. 2012 3;17(3):402–3. 22129037 10.1111/j.1365-3156.2011.02930.x

[53] de Vlas SJ, Gryseels B, van Oortmarssen GJ, Polderman AM, Habbema JD. A pocket chart to estimate true Schistosoma mansoni prevalences. Parasitol Today. 1993 8;9(8):305–7. 10.1016/0169-4758(93)90132-Y 15463790

